# Phenotypic and genetic analysis of cognitive performance in Major Depressive Disorder in the Generation Scotland: Scottish Family Health Study

**DOI:** 10.1038/s41398-018-0111-0

**Published:** 2018-03-13

**Authors:** Joeri J. Meijsen, Archie Campbell, Caroline Hayward, David J. Porteous, Ian J. Deary, Riccardo E. Marioni, Kristin K. Nicodemus

**Affiliations:** 1 Centre for Genomic and Experimental Medicine, Institute of Genetics and Molecular Medicine, University of Edinburgh, Edinburgh, UK; 2 Centre for Cognitive Ageing and Cognitive Epidemiology, University of Edinburgh, Edinburgh, UK; 3 MRC Human Genetics Unit, MRC Institute of Genetics and Molecular Medicine, University of Edinburgh, Edinburgh, UK; 4 Department of Psychology, University of Edinburgh, Edinburgh, UK

## Abstract

Lower performances in cognitive ability in individuals with Major Depressive Disorder (MDD) have been observed on multiple occasions. Understanding cognitive performance in MDD could provide a wider insight in the aetiology of MDD as a whole. Using a large, well characterised cohort (*N* = 7012), we tested for: differences in cognitive performance by MDD status and a gene (single SNP or polygenic score) by MDD interaction effect on cognitive performance. Linear regression was used to assess the association between cognitive performance and MDD status in a case-control, single-episode–recurrent MDD and control-recurrent MDD study design. Test scores on verbal declarative memory, executive functioning, vocabulary, and processing speed were examined. Cognitive performance measures showing a significant difference between groups were subsequently analysed for genetic associations. Those with recurrent MDD have lower processing speed versus controls and single-episode MDD (*β = ***−**2.44, *p* = 3.6 × 10^−04^; *β = ***-**2.86, *p* = 1.8 × 10^−03^, respectively). There were significantly higher vocabulary scores in MDD cases versus controls (*β* = 0.79, *p* = 2.0 × 10^−06^), and for recurrent MDD versus controls (*β* = 0.95, *p*** = **5.8 × 10^−05^). Observed differences could not be linked to significant single-locus associations. Polygenic scores created from a processing speed meta-analysis GWAS explained 1% of variation in processing speed performance in the single-episode versus recurrent MDD study (*p* = 1.7 × 10^−03^) and 0.5% of variation in the control versus recurrent MDD study (*p* = 1.6 × 10^−10^). Individuals with recurrent MDD showed lower processing speed and executive function while showing higher vocabulary performance. Within MDD, persons with recurrent episodes show lower processing speed and executive function scores relative to individuals experiencing a single episode.

## Introduction

Major Depressive Disorder (MDD) is common mental disorder affecting at least 1 in 10 in the United Kingdom^1^ and is a leading cause of disability worldwide. Showing a SNP-based heritability of ~30%^2,3^ and a twin-based estimate of ~40%^4^, MDD has a substantial genetic component. It has been shown that individuals suffering from MDD show lower performance in cognitive domains such as executive function (EF), memory, language and attention^5–7^. The identification and quantification of lower cognitive performance in MDD could lead to a better understanding of the underlying aetiology of depression, to improve treatment of patients, or as an endophenotype for subsequent studies investigating the genetic architecture of MDD. These targeted approaches could possibly lay the groundwork to improve the mental health of MDD patients and therefore lower the burden MDD has on society.

Despite the high prevalence of MDD, cognitive lower scores in MDD have not been as widely studied as in other psychiatric disorders such as bipolar disorder^8^ and schizophrenia^8,9^. Snyder et al.^5^ performed an extensive and the largest-to-date meta-analysis of cognitive performance in MDD, focussing mainly on tasks that measure executive function (EF) with the exception of two non-EF tests measuring vocabulary (language) and digit symbol substitution (processing speed, but is also considered by some to be a component of EF). They observed that MDD patients showed a lower performance in phonemic verbal fluency and digit symbol measures. That is, MDD patients produced significantly fewer words than healthy control individuals and recoded significantly fewer symbols to digits in digit symbol measures. Vocabulary performance was observed to be lower in MDD patients; however, the effect was not significant. Logical memory (LM) immediate and delayed (both measuring verbal declarative memory) have been less well studied compared to other cognitive measures in depression. Lim et al.^6^ conducted the largest meta-analysis study of LM to date (*N* logical memory immediate = 291; *N* logical memory delayed = 348). They observed that MDD patients performed significantly less well than controls on both LM immediate and delayed. This result has been previously reported by smaller studies not included in the Lim et al. study^10,11^, with one exception^12^. Significant lower performances were also observed in the attention domain^6^, via the digit span test and continuous performance test where MDD patients performed slower compared to controls. The final domain examined, visuospatial processing (immediate and delayed visual memory), showed no differences between MDD patients and controls^6^.

As the genomic underpinnings of MDD are poorly understood^13^, we examined genomic associations with cognitive differences as observed in our study as an endophenotype strategy. Using the extensively phenotyped Generation Scotland Cohort Study, we sought to: (a) investigate whether cognitive ability in MDD patients differs from controls without MDD or reported mental illness, (b) assess whether cognitive performance differs between single-episode MDD versus recurrent MDD, (c) investigate cognitive performance between controls and recurrent MDD and (d) to reduce multiple testing we performed genome-wide single locus, genome-wide single-locus interaction, polygenic and polygenic interaction analyses only on cognitive performance tests showing a significant difference within study designs. This study represents the largest single cohort study investigating the association of cognitive performance in depression using a formal clinical diagnosis of MDD and incorporating genomic association analyses. The largest single cohort study investigating cognitive performance in depression is the UK Biobank cohort study^7^ however that study relies on self-reported MDD status and does not examine genetic factors.

## Materials and methods

### Cohort data and phenotyping

Generation Scotland: the Scottish Family Health Study (GS:SFHS) is a family-based cohort study sampled from the general population in Scotland (www.generationscotland.org)^14,15^. The study design has been widely documented^14,15^. In short, between 2006 and 2011 over 24,000 subjects were recruited into the study. The initial sample of study subjects (*N* = 7953) were registered with general medical practitioners, between 35 and 65 years, and from five regions of Scotland. These initial study subjects were asked to bring a relative within the age range 18–99 to the baseline data collection. Participants were asked to fill in health, lifestyle and family history questionnaires and answer a 30 min interview which included questions about possible mental ill health. If participants answered positively on either of the 2 mental health screening questions (“Have you ever seen anybody for emotional or psychiatric problems?” and “Was there ever a time when you, or someone else, thought you should see someone because of the way you were feeling or acting?”) (*N* = 4539), they were asked to take part in a Structured Clinical Interview for DSM-IV (SCID)^16^, focussing on mood disorders. Individuals answering “no” to both questions were assigned to the control group. Individuals who completed the SCID but did not meet the criteria for MDD or bipolar disorder were subsequently assigned to the control group^17^ (*N* = 1727). Finally, individuals who were invited for the SCID interview but refused to take part (*N* = 507) were not assigned to either case or control group^3^.

Four cognitive domains were measured in Generation Scotland: processing speed (Wechsler Digit Symbol Substitution Test; recoding symbols to digits^18^—DST), verbal declarative memory (Wechsler Logical Memory Test; sum of immediate and delayed recall of an oral story^19^—LM1 and LM2), executive functioning (the phonemic verbal fluency test; using the letters C, F, and L, each for one minute^20^—VFT), language (the Mill Hill Vocabulary Scale, Junior and Senior Synonyms combined—finding a synonym of a given word^21^—MHVS) and the difference between logical memory immediate and delayed (LM1–LM2). The correlation between scores on tests of these different cognitive domains are reported in Supplementary Tables S1-S4.

In addition to age and sex, we selected lifestyle factors (self-reported smoking and alcohol intake), socioeconomic status (the Scottish Index of Multiple Deprivation^22^), medication usage (anti-depressants and mood stabilisers) and 15 genetic principal components to control for population stratification. These variables have been previously used as covariates in Cullen et al. 2015^7^ to investigate cognitive differences in depression using the UK Biobank cohort.

### Genetic data

DNA of 20,128 GS:SFHS participants was analysed by means of high density genome-wide bead array genotyping (Illumina OmniExpress 700K SNP GWAS and 250K exome chip). We selected a set of unrelated individuals for use in our analyses, to remove the influence of shared environments. We removed single nucleotide polymorphisms (SNPs) and individuals with a missingness of >1% and removed rare SNPs with a minor allele frequency <0.01 leaving 557,292 SNPs for analysis. We used Genome-wide Complex Trait Analysis^23^ to extract a list of genetically unrelated individuals from a predefined list of participants with a known MDD SCID diagnosis or controls. Seven thousand, one hundred and seventy-two unrelated individuals (relatedness<0.025, corresponding to second degree cousins) were selected, of which 1042 individuals (14.5%) were diagnosed with either single or recurrent depression. One hundred and five individuals were removed due to the lack of self-reported medical background information. Another 25 individuals with self-reported Alzheimer’s and/or Parkinson’s disease were removed leading to a total of 7012 individuals, of which 1021 individuals (14.5%) were diagnosed with a form of depression.

### Statistical analysis

#### Phenotypic differences

We used phi coefficients and Spearman correlation coefficients to determine the level of correlation between the pool of potential covariates and MDD case-control, single-recurrent or control-recurrent status. As a continuous variable, age was assessed using the Spearman correlation coefficient. As all other variables were binary, their correlations were assessed using the phi coefficient, with associated *p*-values from either a *χ*^2^ or Fisher’s exact test. The Fisher’s exact test was used when observed cell counts in the 2 × 2 contingency table were <5. No potential covariate was strongly correlated with MDD case-control (Supplementary Table S5), single-recurrent (Supplementary Table S6) or control-recurrent (Supplementary Table S7) status aside from age, sex and medication usage in the case-control study and solely medication usage in both the single-recurrent and control-recurrent MDD study, as expected. To keep in line with Cullen et al., 2015 all covariates (sex, age, alcohol consumption, smoking tobacco, medication usage, socioeconomic status and 15 principal components) were included in the full model.

Multiple regression analysis was performed for each cognitive test and the diagnosis label before and after controlling for covariates. We used the following models: a baseline model (1):

1 $$\mathrm {Cognitive}\,\mathrm {ability}\,\mathrm {test}_k = \beta _0 + \beta _{\mathrm {diagnosis}\,\mathrm {label}}\mathrm {diagnosis}\,\mathrm {label}$$

and a full model (2):

2 $$\begin{array}{ccccc}\\ \mathrm {Cognitive}\,\mathrm {ability}\,\mathrm {test}_k = & \beta _0 + \beta _{\mathrm {diagnosis}\,\mathrm {label}}\mathrm {diagnosis}\,\mathrm {label}\\\\ & + \mathop {\sum}\limits_{i = 1}^n {\beta _i\,\mathrm {Covariates}_i} \\ \end{array}$$

We observed that medication usage contained many missing values (52%), with only a small percentage of all participants answering positively (5.1%). Therefore, we performed model 2 and all subsequent analyses twice (1) including medication usage (M2A) and (2) excluding medication usage (M2B) as a covariate. A Bonferroni significance level of *p* < 8.3 × 10^−03^ (*p* = 0.05/6 cognitive tests) was used. All models were run using the R Statistical Computing Environment^24^ v 3.1.0.

#### Single-Locus analysis

We performed a Genome-Wide Association Study (GWAS) for the cognitive performance variables that showed a significant difference in the phenotypic analyses. We further tested whether each SNP’s association with cognitive performance depended on MDD status via a Genome-Wide by Environment Interaction Study (GWEIS). The GWAS analyses can be seen as a baseline model and GWEIS as a measure of non-additive effects for SNP and depression case status. The standard Bonferroni significance level of *p* < 5 × 10^−08^ is conservative, as many SNPs are in linkage disequilibrium thus statistical tests are not independent. Therefore, we applied a less conservative significant level *p* < 1.52 × 10^−07^ derived from the Genetic type 1 Error Calculator (GEC)^25^. All models were run using PLINK version v1.90b1g.

#### Polygenic analysis

**Fig. 1:**
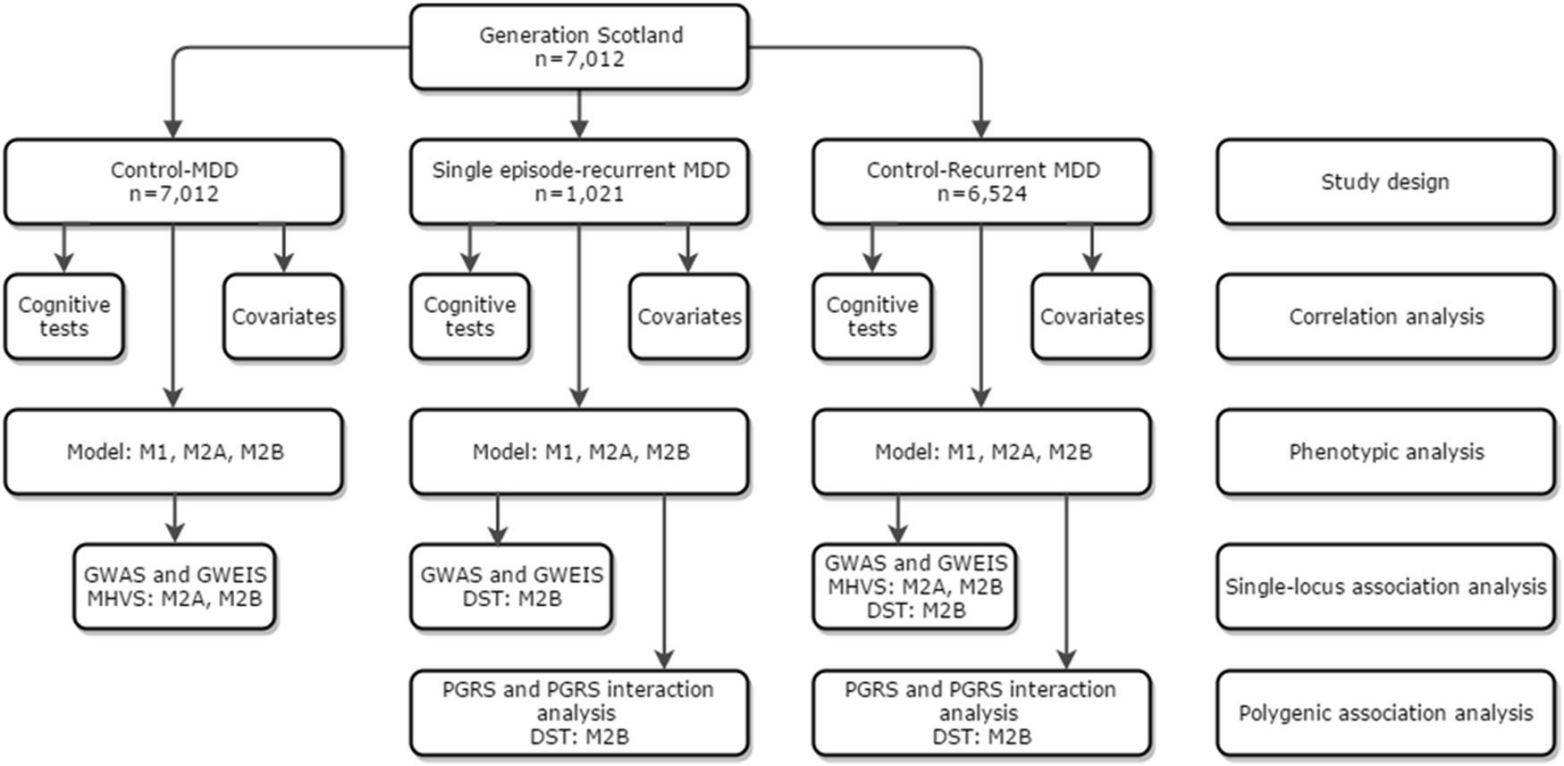
M1 = no covariates, M2A = controlling for all covariates and M2B = controlling for all covariates except medication

Polygenic Risk Scores (PGRS) were calculated for five* p*-value threshold ranges (0–0.01, 0–0.05, 0–0.1, 0–0.5, 0–1) using summary output from the Cohorts for Heart and Aging Research in Genomic Epidemiology (CHARGE) meta-analysis GWAS of DST and similar tests that controlled for sex, age, assessment centre, education and community^26^. Generation Scotland is a part of the CHARGE consortium but was not included in this specific meta-GWAS study. The CHARGE consortium performed a sample size weighted meta-analysis because of the differences in the test methodology and measurement units. The *z*-statistic was weighted by the effective sample size (sample size × (observed dosage variance/expected dosage variance)) for each SNP. We pruned the Generation Scotland dataset for linkage disequilibrium (window size = 50 kb, step size = 5 kb and *r*^2^ threshold = 0.25) and converted the CHARGE *z*-statistics to standardised beta coefficients using the *z*-score and standard error provided by CHARGE. We performed a linear regression model between the DST and the polygenic risk scores as well as a model including polygenic risk score-by-MDD status interaction in a controls-recurrent MDD and single-recurrent MDD study. Consistently with previous analyses, we restricted our polygenic score analysis to the groups where we had observed significant differences. We controlled for all covariates and the number of valid genotypes in a model that did not include medication usage. Figure 1 shows a graphic representation of performed analyses.

## Results

### Descriptive statistics

We observed significant differences in the distributions of sex, age, alcohol consumption, smoking tobacco, medication usage and socioeconomic status across MDD status with a higher frequency of females (69–72%), tobacco smokers (23–26.8%) and medication users in the MDD case group (Table 1). Within MDD cases, alcohol drinkers represented a significant lower frequency in the recurrent MDD (83.5%) group than the single-episode MDD (88.8%) but a higher frequency in medication usage. On average, controls were slightly but significantly older than cases with MDD and lived in less deprived areas.

**Table 1:** Demographics and medical history by MDD case status

	MDD Status		
	Control	Single	Recurrent
Covariate	*N* = 5991	*N* = 488	*N* = 533
Sex (*N*, % Female, 0 NA)	3252 (54)	336 (69)	384 (72)*
Age (M, SD, 0 NA)	51.7 (13.8)	49.1 (12.6)	50.1 (11.1)*
Alcohol (*N*, % Drinking, 107 NA)	5410 (91.6)	424 (88.8)	437 (83.5)
Smoking (*N*, % Smoking, 90 NA)	837 (14.2)	111 (23)	141 (26.8)*
Medication (*N*, % Using, 3595 NA)	73 (2.49)	31 (12.4)	71 (29.8)
SES (M, SD, 363 NA)	4080 (1819.3)	3836 (1853.1)	3422.7 (1966.4)

*All association show significant group differences at 0.05, corrected for multiple testing except for single-episode versus recurrent MDD

### Cognitive association by depression status

We performed three linear regression analyses for each cognitive test (the dependent variable). The predictor variable was MDD diagnosis, classified as either control-MDD, single-episode–recurrent MDD or control-recurrent MDD. No other covariates were considered in these baseline models (Table 2). No significant association was observed between MDD and cognitive test scores, except for digit symbol substitution in the single-episode–recurrent comparison (*β* = −3.41, *p* = 5.8 × 10^–04^), with the recurrent MDD group recode fewer symbols to digit compared to single-episode MDD group.

**Table 2:** Association between diagnosis label and cognitive performance in both study designs, without controlling for covariates

	Control-MDD			Single-Recurrent MDD			Control-Recurrent MDD		
	*β*	Pr(>|t|)	*N*	*β*	Pr(>|t|)	*N*	*β*	Pr(>|t|)	*N*
LM1	0.20	0.12	6974	−0.56	0.01	1021	−0.06	0.72	6486
LM2	0.21	0.13	6936	−0.52	0.03	1016	−0.03	0.86	6452
LM1–LM2	−0.03	0.59	6936	−0.02	0.86	1016	−0.04	0.61	6452
DTS	−0.02	0.96	6936	**−3.41**	**5.8E−04**	**1011**	−1.65	0.02	6452
VFT	0.03	0.93	6934	−0.03	0.96	1019	0.01	0.97	6447
MHVS	0.05	0.75	6887	0.26	0.37	1013	0.17	0.41	6401

Bolded results are significant after Bonferroni correction

*DST* digit symbol substitution test, *LM1* Logical memory immediate, *LM2* logical memory delayed, *MHVS* Mill Hill vocabulary score, *VFT* verbal fluency total

We then performed linear regression on the full model, including all covariates that were used in Cullen et al.^7^, which includes medication usage (Supplementary Table S8). We observed a significant difference after correcting for multiple testing in the MHVS in both the control-MDD and control-recurrent MDD study. Individuals with depression had higher scores in the MHVS, identifying on average 0.66 more synonyms relative to controls (*β = *0.66, *p = *2.96 × 10^−03^). Between controls and individuals with recurrent MDD, participants with recurrent depression performed even higher, with 1.07 more synonyms identified (*p* = 6.0 × 10^−04^).

When leaving out medication usage (Table 3) we observed the same significant higher performance of the MHVS in the MDD and recurrent MDD group in the control-MDD *(β = *0.79, *p = *2.02 × 10^−06^) and control-recurrent MDD (*β = *0.95, *p = *5.8 × 10^−05^) study design. Individuals with recurrent MDD recoded significantly fewer symbols back to digits compared to their study design counterparts in the single-episode–recurrent (*β = *−2.86, *p = *1.8 × 10^−03^) and control-recurrent MDD (*β = *−2.44*, p = *3.6 × 10^−04^) study designs

**Table 3:** Association between diagnosis label and cognitive performance in both study designs, after controlling for all covariates except medication

	Control-MDD			Single-Recurrent MDD			Control-Recurrent MDD		
	*β*	Pr(>|t|)	*N*	*β*	Pr(>|t|)	*N*	*β*	Pr(>|t|)	*N*
LM1	0.19	0.18	6447	−0.41	0.09	923	−5.0E−03	0.97	6008
LM2	0.15	0.31	6410	−0.36	0.16	918	−0.02	0.88	5975
LM1–LM2	0.01	0.89	6410	−0.03	0.80	918	6.3E−03	0.95	5975
DST	−1.09	0.03	6411	**−2.86**	**1.8E−03**	**913**	**−2.44**	**3.6E−04**	**5976**
VFT	0.89	0.04	6417	0.30	0.69	921	1.04	0.06	5979
MHVS	**0.79**	**2.02E−06**	**6372**	0.42	0.13	916	**0.95**	**5.8E−05**	**5935**

Bolded results are significant after Bonferroni correction

DST digit symbol substitution test, LM1 Logical memory immediate, LM2 logical memory delayed, MHVS Mill Hill vocabulary score, VFT verbal fluency total

### Polygenic score analysis

In the single-episode–recurrent study design, the DST PGRS was significantly associated with DST performance at all but two *p-*value thresholds (Bonferroni *p* = 0.01; 0.05/5 PGRS ranges), indicating that the DST polygenic risk score explained a significant amount of variation (most significant polygenic score: *R*^2^ 1%, *p*-value threshold = 0.1, *p* = 1.66 × 10^−03^) in performance among MDD cases (Table 4). We observed significant statistical association with each PGRS range in the control-recurrent MDD study design with the PGRS explaining between 0.13 and 0.5% of variation (Table 4). However, the effect of the DST polygenic score did not differ between single-episode–recurrent cases nor between controls and recurrent MDD cases. We did not observe a PGRS by MDD group interaction on DST performance (Supplementary Table S9).

**Table 4:** Association between DST performance and PGRS derived from the DST meta-analysis of the CHARGE consortium

		Single-Recurrent MDD			Control-Recurrent MDD	
Range	Direction	Pr(>|t|)	*R*^2^ (%)	Direction	Pr(>|t|)	*R*^2^ (%)
0–0.01	+	0.14	0.48	**+**	**1.63E−03**	**0.13**
0–0.05	**+**	**4.75E−03**	**0.85**	**+**	**9.95E−06**	**0.23**
0–0.1	**+**	**1.66E−03**	**1**	**+**	**5.12E−08**	**0.36**
0–0.5	+	0.011	0.66	**+**	**7.83E−10**	**0.46**
0–1	**+**	**8.42E−03**	**0.7**	**+**	**1.61E−10**	**0.5**

Bolded results are significant after Bonferroni correction

### Single-locus analysis

GWAS (Supplementary Figure S1a-b) and GWEIS (Supplementary Figure S2a-b) analyses was performed on MHVS in the control-MDD and control-recurrent MDD study designs excluding medication usage. No SNP was observed below the GEC significance threshold in the MHVS analyses (GEC *p = *1.52* × *10^−07^). The same analysis was performed for DST in the single-episode–recurrent and control-recurrent MDD study designs without controlling for medication usage (Supplementary Figures S3a-b and S4a-b). We did not observe a significant association between genomic variation and DST. Both the strongest non-significant GWAS and GWEIS hit were associated with digit symbol performance and observed in the single-episode–recurrent MDD study design. SNP rs10829637 (*p* = 3.3 × 10^−07^) located on chromosome 10 in *LOC107984280* was the most significant GWAS hit and rs911684 (*p* = 6.7 × 10^−07^) located on chromosome 14 in *LOC100506999* was the most significant GWEIS hit. Other GWAS and GWEIS results can be found in Supplementary Figure S5a-b, S6a-b.

## Discussion

This study of cognitive performance in MDD is the largest single cohort study with a formal clinical diagnosis of MDD and incorporating genomic association analyses. The only larger single cohort study is UK Biobank, which does not contain a formal clinical diagnosis of MDD and did not investigate genetics association. Moreover, the cognitive battery used in Generation Scotland is standardised and validated on large representative samples using pre-existing evidence while the cognitive battery used in UK Biobank was bespoke and designed for UK Biobank itself.

We observed significantly higher MHVS scores in MDD cases versus controls, and between recurrent depression versus controls with and without controlling for medication usage, with’cases’ performing higher than the latter in both studies. The same directionality of effect was observed in UKB by Cullen et al.^7^; they also observed a significant higher score in vocabulary performance in MDD case groups compared to controls. We also observed significant lower performance of DST between recurrent and single-episode MDD cases, and between recurrent MDD and controls; however, in this case the ‘cases’ performed less well in both study designs. We also observed a significant amount of variation explained in DST performance using the CHARGE consortium DST polygenic risk score; however, this result was observed across cases and controls and did not differ by case status, indicating that the DST polygenic risk score may not be a useful endophenotype for depression.

Our results are consistent with the largest meta-analysis of case-control differences in digit symbol coding performance, which found that individuals with depression performed significantly lower than controls^4^. One previous study not included in the recent meta-analysis^4^ examining differences in digit symbol coding performance between individuals with depression (current (*N* = 37) or previous (*N* = 81)) and controls (*N* = 50) found no significant difference between the three groups, but the sample size was modest^27^. We also report no significant differences between cases and controls or single-episode versus recurrent MDD on vocabulary, also consistent with Snyder et al^4^. However, we were unable to replicate some results previously reported in the literature^5–7,10–12,28,29^. Snyder et al.^4^ observed significant lower performance in phonemic verbal fluency between cases and controls whereas we observed no significant difference. One possible reason is through the inclusion of people in the control group that have symptoms of depression but do not meet the criteria of being diagnosed with MDD, in other words, misclassification of controls, which may have biased our estimates toward the null. Misclassification of controls as MDD participants might be possible due to the screening questions: “*Have you ever seen anybody for emotional or psychiatric problems?*” and “*Was there ever a time when you, or someone else, thought you should see someone because of the way you were feeling or acting?*”. However, this is unlikely due to the subsequent SCID interview given by a trained clinical nurse. Given that this interview was given to all MDD cases in GS:SFHS, misclassification would be less likely between single-episode MDD versus recurrent MDD. Second, publication bias could have influenced results from meta-analyses. Our sample size, although the second largest to investigate MDD and cognition to date, may be underpowered to detect small differences in cognitive performance. Although we removed individuals with Alzheimer and Parkinson’s disease and controlled for smoking and alcohol intake, we did not control for other disorders that may affect cognition. Many previous studies focused on clinical populations, whereas our study is population based; clinical populations may have more severe forms of depression. The use of simpler models in meta-analyses, which do not control for covariates, may obscure signals. Finally, observed cognitive performance in MDD in the literature are mainly observed in large meta-analyses which increases the study heterogeneity, while our results are derived from a much more homogeneous single cohort study. However, both Snyder et al.^4^ and Lim et al.^5^ observed significant heterogeneity and subsequently applied random-effects meta-analytic models that do not assume homogeneity of effect between studies. We also were not able to assess all cognitive domains which could show signs of cognitive impairments in MDD, such as visuospatial processing and attention^6^. Finally, we were unable to control for the effects of antidepressant use on cognitive performance in the full sample, which may lead to poorer performance in cases^9^.

Cognitive differences between single-episode and recurrent MDD have been not as well studied as differences between MDD cases and controls^30,31^. Talarowska et al.^30^ compared the cognitive performance of 210 patients with MDD (single-episode *N* = 60, recurrent *N* = 150) and observed that the cognitive domains of executive functioning, memory and processing speed showed significant lower performance in recurrent MDD in relation to single-episode MDD. The largest study to date to assess cognitive differences between single-episode and recurrent depression has been the UK Biobank study^7^. Cullen et al. (2015) observed higher performance in single-episode MDD vs controls (numeric and prospective memory), however moderate and severe MDD groups performed lower (e.g. reaction time and numeric memory) compared to both the single MDD and control group.

Cullen et al. 2015 observed the same counter-intuitive higher performance in vocabulary for MDD cases compared to controls and provided several possible explanations for this. It may include differential selection (depressed individuals are more likely to participate than controls), differential recall (cognitive test is associated with greater recall), higher health literacy (individuals with a higher intelligence are quicker to spot possible health issues and therefore quicker to see a GP) or residual confounding.^7^ As vocabulary is a crystallised intelligence measure where the tests demand recall ability, and as we observed the same higher performance in a second large population-based cohort, we hypothesise that differential recall and higher health literacy are the most plausible explanations.

That we did not observe a significant genome-wide hit for MDD was unsurprising as it is a clinically heterogeneous disorder with multiple SNPs of small effect, which would be difficult to observe without very large sample sizes. We controlled for LD structure in GWAS/GWEIS by applying a less conservative GEC significance threshold which takes into account LD between SNPs. We compared *p*-values of SNPs associated with depression in a large cohort study^32^ with our results from the GWEIS studies (Supplementary Table S10). Four SNPs overlapped with those available in Generation Scotland and for 18 SNPs we used 52 proxy SNPs (*r*^2^ > 0.8). We observed a consistent positive association with *p*-value <0.05 for the GWEIS of MHVS (both case-control and control-recurrent) and for the GWEIS of DST in control-recurrent analyses for SNP rs4143229 which is intronic and located in *ENOX1*. A recent GWAS of antidepressant treatment response at 12 weeks to selective serotonin reuptake inhibitors (SSRIs) showed suggestive association with another intronic SNP in *ENOX1*, rs17538444^33^. Using Quanto^34^ for gene-by-environment power calculations, setting *α* = 0.05, two-sided, and using a MAF of 0.5 (as our top SNP had a MAF of 0.48), and observed MDD proportion and distribution of DSST, we concluded that a sample size of 2885 individuals was required to detect an interaction effect at 80% power. Although a significant amount of variation in DST was explained by the CHARGE consortium DST polygenic score, it was not specific to MDD cases and the effect did not vary by MDD case status. Polygenic scores often explain only a small amount of variation in endophenotypes. In this study, we looked for main and polygenic effects; it might be possible that more variation can be explained by incorporating possible genetic interactions between loci and/or the environment or interactions of two or more loci.

The main strength of this study is that is has assessed the association between MDD and cognitive ability in a large homogeneous population sample, using standardised tests and outcome measures across all participants. This represents a significant advantage over previous studies that used either meta-analytic (combination of effects across studies) or mega-analytic (combining individual-level data across studies) methods to improve statistical power. The division of the dataset in three study designs based on MDD diagnosis allowed us to assess cognitive performance based on MDD severity. Limitations of this study are the sample size (*N* = 7012) which results in a low powered interaction analysis, underreporting of antidepressant and mood stabiliser medication usage (<40%) and finally certain cognitive domains are not measured in the Generation Scotland cognitive battery, i.e., visuospatial perception.

In conclusion, we have shown that cognitive performance in some domains significantly differs between controls and MDD groups but also within MDD groups. This difference could not be linked to single-locus associations but a small proportion of variation could be explained by means of a polygenic approach.

## Electronic supplementary material

MeijsenJ_SupplementaryMaterials
