# Supplementary material for: Phenotypic and genetic analysis of cognitive performance in Major Depressive Disorder in the Generation Scotland: Scottish Family Health Study
Source: Transl Psychiatry. 2018 Mar 13;8:63. doi: 10.1038/s41398-018-0111-0 (PMC5847617; doi:10.1038/s41398-018-0111-0)
Supplement: Supplementary file 1 — MeijsenJ_SupplementaryMaterials [file 41398_2018_111_MOESM1_ESM.docx]

**Table S1: Correlation (*p*-values) between cognitive performance tests for controls**

|  | LM1 | DST | VFT | MHVS | LM2 | LM1-LM2 |
| --- | --- | --- | --- | --- | --- | --- |
| LM1 | 1 |  |  |  |  |  |
| DST | 0.29 (< 2.2E-16) | 1 |  |  |  |  |
| VFT | 0.15 (< 2.2E-16) | 0.25 (< 2.2E-16) | 1 |  |  |  |
| MHVS | 0.23 (< 2.2E-16) | 0.08 (7.01E-10) | 0.39 (< 2.2E-16) | 1 |  |  |
| LM2 | 0.86 (< 2.2E-16) | 0.30 (< 2.2E-16) | 0.15 (< 2.2E-16) | 0.21 (< 2.2E-16) | 1 |  |
| LM1-LM2 | 0.07 (5.91E-08) | -0.08 (2.34E-10) | -0.04 (7.1E-04) | -0.008 (0.5) | -0.44 (< 2.2E-16) | 1 |

Correlations between continuous variables were calculated using the Pearson correlation coefficient. LM = logical memory, DST = digit symbol test, VFT = verbal fluency test, MHVS = Mill-Hill Vocabulary test.

**Table S2: Correlation (*p*-values) between cognitive performance tests for MDD cases**

|  | LM1 | DST | VFT | MHVS | LM2 | LM1-LM2 |
| --- | --- | --- | --- | --- | --- | --- |
| LM1 | 1 |  |  |  |  |  |
| DST | 0.34 (< 2.2E-16) | 1 |  |  |  |  |
| VFT | 0.22 (5.5E-13) | 0.25 (< 2.2E-16) | 1 |  |  |  |
| MHVS | 0.27 (< 2.2E-16) | 0.11 (4.1E-04) | 0.39 (< 2.2E-16) | 1 |  |  |
| LM2 | 0.86 (< 2.2E-16) | 0.35 (< 2.2E-16) | 0.22 (1.1E-12) | 0.25 (< 2.2E-16) | 1 |  |
| LM1-LM2 | 0.15 (5.3E-07) | -0.07 (2.4E-02) | -0.02 (0.43) | 0.01 (0.7) | -0.35 (< 2.2E-16) | 1 |

Correlations between continuous variables were calculated using the Pearson correlation coefficient. LM = logical memory, DST = digit symbol test, VFT = verbal fluency test, MHVS = Mill-Hill Vocabulary test.

**Table S3: Correlation (*p*-values) between cognitive performance tests for single episode MDD cases**

|  | LM1 | DST | VFT | MHVS | LM2 | LM1-LM2 |
| --- | --- | --- | --- | --- | --- | --- |
| LM1 | 1 |  |  |  |  |  |
| DST | 0.31 (1.2E-12) | 1 |  |  |  |  |
| VFT | 0.20(5.8E-06) | 0.22(5.4E-07) | 1 |  |  |  |
| MHVS | 0.24 (3.6E-08) | 0.05 (0.27) | 0.39 (< 2.2E-16) | 1 |  |  |
| LM2 | 0.85 (< 2.2E-16) | 0.29 (5.3E-11) | 0.2 (1.4E-05) | 0.24 (1.1E-07) | 1 |  |
| LM1-LM2 | 0.20 (7.5E-06) | 0.01 (0.8) | -0.01 (0.8) | 0.02 (0.7) | -0.34 (2.4E-14) | 1 |

Correlations between continuous variables were calculated using the Pearson correlation coefficient. LM = logical memory, DST = digit symbol test, VFT = verbal fluency test, MHVS = Mill-Hill Vocabulary test.

**Table S4: Correlation (*p*-values) between cognitive performance tests for recurrent episode MDD cases**

|  | LM1 | DST | VFT | MHVS | LM2 | LM1-LM2 |
| --- | --- | --- | --- | --- | --- | --- |
| LM1 | 1 |  |  |  |  |  |
| DST | 0.35 (2.26E-16) | 1 |  |  |  |  |
| VFT | 0.24 (2E-08) | 0.28 (5.4E-11) | 1 |  |  |  |
| MHVS | 0.3 (< 3.4E-12) | 0.17 (6.7E-05) | 0.41 (< 2.2E-16) | 1 |  |  |
| LM2 | 0.87 (< 2.2E-16) | 0.4 (< 2.2E-16) | 0.24 (1.8E-08) | 0.27 (< 1.88E-10) | 1 |  |
| LM1-LM2 | 0.12 (6E-03) | -0.14 (9.6E-04) | -0.04 (0.41) | 0.007 (0.9) | -0.38 (< 2.2E-16) | 1 |

Correlations between continuous variables were calculated using the Pearson correlation coefficient. LM = logical memory, DST = digit symbol test, VFT = verbal fluency test, MHVS = Mill-Hill Vocabulary test.

**Table S5: Correlation (*p*-values) between covariates for MDD cases and controls**

|  | Control-MDD | Sex | Age | Alcohol | Smoking | Medication | SES |
| --- | --- | --- | --- | --- | --- | --- | --- |
| Control-MDD | 1 |  |  |  |  |  |  |
| Sex | 0.12 (5.2E-22) | 1 |  |  |  |  |  |
| Age | -0.054 (5.0E-6) | -0.06 (3.2E-6) | 1 |  |  |  |  |
| Alcohol | -0.07 (3.23E-08) | -0.05 (8.44E-05) | -0.05 (1.54E-05) | 1 |  |  |  |
| Smoking | 0.11 (2.49E-18) | -0.03 (0.03) | -0.15 (1.04E-37) | -0.04 (1.83E-03) | 1 |  |  |
| Medication | 0.29 (1.37E-64) | 0.05 (3.0E-03) | 0.04 (8.0E-03) | -0.06 (1.09E-03) | 0.07 (9.42E-05) | 1 |  |
| SES | -0.08 (1.66E-11) | -0.05 (1.68E-05) | 0.14 (5.62E-32) | 0.08 (6.85E-11) | -0.19 (1.78E-56) | -0.07 (5.52E-06) | 1 |

Correlations between binary variables were calculated using the phi coefficient and corresponding χ^2^ or Fisher’s exact test *p*-value. Fisher’s exact test was used when cell sizes in the 2 x 2 contingency table with observed values smaller than 5. Correlations between continuous variables were calculated using the Spearman correlation coefficient. SES = socioeconomic status.

**Table S6: Correlation (*p*-values) between covariates for single and recurrent MDD**

|  | Single-Recurrent | Sex | Age | Alcohol | Smoking | Medication | SES |
| --- | --- | --- | --- | --- | --- | --- | --- |
| Single-Recurrent | 1 |  |  |  |  |  |  |
| Sex | 0.03 (0.29) | 1 |  |  |  |  |  |
| Age | 0.04 (0.18) | -0.06 (0.06) | 1 |  |  |  |  |
| Alcohol | -0.08 (4.80E-306) | 0.04 (0.2) | -0.019 (0.54) | 1 |  |  |  |
| Smoking | 0.04 (0.19) | -0.01 (0.76) | -0.18 (1.47E-09) | -0.1 (0.002) | 1 |  |  |
| Medication | 0.21 (3.79E-06) | -0.03 (0.61) | 0.15 (8.5E-04) | -0.08 (0.11) | 0.04 (0.47) | 1 |  |
| SES | -0.10 (0.001) | -0.06 (0.03) | 0.17 (5.01E-08) | 0.06 (0.06) | -0.22 (1.54E-12) | -0.04 (0.33) | 1 |

Correlations between binary variables were calculated using the phi coefficient and corresponding χ^2^ or Fisher’s exact test *p*-value. Fisher’s exact test was used when cell sizes in the 2 x 2 contingency table with observed values smaller than 5. Correlations between continuous variables were calculated using the Spearman correlation coefficient. SES = socioeconomic status.

**Table S7: Correlation (*p*-values) between covariates for recurrent MDD cases and controls**

|  | Control-Recurrent | Sex | Age | Alcohol | Smoking | Medication | SES |
| --- | --- | --- | --- | --- | --- | --- | --- |
| Control-  Recurrent MDD | 1 |  |  |  |  |  |  |
| Sex | -3.44E-04 (0.977) | 1 |  |  |  |  |  |
| Age | -0.03 (-0.03) | 0.29 (7.92E-130) | 1 |  |  |  |  |
| Alcohol | -1.94E-3 (0.87) | 0.15 (1.47E-35) | 0.25 (1.98E-92) | 1 |  |  |  |
| Smoking | 6.54E-03 (0.6) | 0.23 (1.45-82) | 0.08 (2.88E-12) | 0.39 (2.18E-234) | 1 |  |  |
| Medication | 2.83E-04 (0.98) | 0.86 (0) | 0.3 (3.27E-140) | 0.16 (1.95E-37) | 0.22 | 1 |  |
| SES | -9.3E-04 (0.94) | 0.07 (2.39E-09) | -0.08 (3.41E-12) | -0.04 (5.15E-04) | -7.54E-03 (0.55) | -0.44 (3.75E-301) | 1 |

Correlations between binary variables were calculated using the phi coefficient and corresponding χ^2^ or Fisher’s exact test *p*-value. Fisher’s exact test was used when cell sizes in the 2 x 2 contingency table with observed values smaller than 5. Correlations between continuous variables were calculated using the Spearman correlation coefficient. SES = socioeconomic status.

**Table S8: Association between diagnosis label and cognitive performance in both study designs, after controlling for all covariates including medication usage**

| Control-MDD | | | Single-Recurrent MDD | | | Control-Recurrent MDD | | |  |  |
| --- | --- | --- | --- | --- | --- | --- | --- | --- | --- | --- |
| β | Pr(>\|t\|) | N | β | Pr(>\|t\|) | N | β | Pr(>\|t\|) | N |  |  |
|  | LM1* | 0.40 | 0.05 | 3172 | -0.28 | 0.44 | 442 | 0.21 | 0.47 | 2947 |
|  | LM2 | 0.31 | 0.17 | 3167 | -0.47 | 0.22 | 442 | -0.02 | 0.92 | 2942 |
|  | LM1-LM2 | 6.6E-02 | 0.56 | 3167 | 0.19 | 0.33 | 442 | 0.19 | 0.22 | 2942 |
|  | DST | -1.15 | 0.13 | 3167 | -1.09 | 0.41 | 440 | -1.84 | 0.08 | 2944 |
|  | VFT | 1.07 | 0.08 | 3160 | 2.36 | 0.04 | 441 | 1.99 | 0.02 | 2936 |
|  | MHVS | **0.66** | **2.96E-03** | **3148** | 0.99 | 0.01 | 442 | **1.07** | **6.0E-04** | **2923** |

*LM1: Logical memory immediate, LM2: logical memory delayed, DST: digit symbol substitution test, VFT: verbal fluency total, MHVS: Mill Hill vocabulary score. Bolded results are significant after Bonferroni correction.

**Table S9. Association between DST performance and PGRS*MDD status derived from the DST meta-analysis of the CHARGE consortium.**

|  | | Single-Recurrent MDD | | Control-Recurrent MDD | |
| --- | --- | --- | --- | --- | --- |
| Range | β | | Pr(>\|t\|) | β | Pr(>\|t\|) |
| 0-0.01 | 2.36E^4^ | | 9.75E^-02^ | 1.10E^-4^ | 0.29 |
| 0-0.05 | 3.15E^4^ | | 0.37 | 3.08E^4^ | 0.24 |
| 0-0.1 | 5.42E^4^ | | 0.31 | 4.95E^4^ | 0.22 |
| 0-0.5 | 2.31E^5^ | | 0.15 | 1.06E^5^ | 0.39 |
| 0-1 | 4.72E^5^ | | 0.11 | 2.21E^5^ | 0.34 |

**Table S10: Comparison between Wray et al, 2017 observed results and Generation Scotland equivalent SNPs.**

|  | | | | GWEIS MHVS CC | GWEIS MHVS CR | GWEIS DST CR | GWEIS DST SR |
| --- | --- | --- | --- | --- | --- | --- | --- |
| SNP  Wray et al, 2017 | p | SNP  Generation Scotland | r | p | p | p | p |
| **rs4143229** | **2.50E-08** | **rs4143229** | **N.A** | **0.0307** | **0.02076** | **0.005283** | **0.176** |
| **rs12552** | **6.10E-19** | **rs12552** | **N.A** | **0.521** | **0.853** | **0.6496** | **0.9415** |
| **rs11643192** | **3.40E-08** | **rs11643192** | **N.A** | **0.2208** | **0.09025** | **0.4155** | **0.09073** |
| **rs1833288** | **2.60E-08** | **rs1833288** | **N.A** | **0.2129** | **0.1569** | **0.32** | **0.9892** |
| rs159963 | 3.20E-08 | rs301806 | 1 | 0.1536 | 0.8932 | 0.4014 | 0.8966 |
| rs159963 | 3.20E-08 | rs301805 | 0.966 | 0.1395 | 0.8448 | 0.3632 | 0.8941 |
| rs159963 | 3.20E-08 | rs4908760 | 0.811 | 0.3446 | 0.8495 | 0.4729 | 0.4594 |
| rs1432639 | 4.60E-15 | rs2012697 | 1 | 0.1228 | 0.1945 | 0.2898 | 0.5964 |
| rs1432639 | 4.60E-15 | rs2568958 | 1 | 0.1259 | 0.1626 | 0.3438 | 0.7348 |
| rs1432639 | 4.60E-15 | rs3101336 | 1 | 0.1473 | 0.1567 | 0.3578 | 0.7346 |
| rs1432639 | 4.60E-15 | rs2815752 | 0.962 | 0.1277 | 0.1642 | 0.3576 | 0.7346 |
| rs1432639 | 4.60E-15 | rs7531118 | 0.8 | 0.1125 | 0.1877 | 0.4689 | 0.9 |
| rs12129573 | 4.00E-12 | rs1160682 | 0.965 | 0.6188 | 0.9106 | 0.1785 | 0.07364 |
| rs12129573 | 4.00E-12 | rs11210201 | 0.932 | 0.7437 | 0.8731 | 0.6114 | 0.4146 |
| rs12129573 | 4.00E-12 | rs1885246 | 0.932 | 0.7527 | 0.8652 | 0.6126 | 0.4004 |
| rs12129573 | 4.00E-12 | rs1475064 | 0.87 | 0.8984 | 0.6614 | 0.4694 | 0.4062 |
| rs12129573 | 4.00E-12 | rs9425120 | 0.864 | 0.9921 | 0.8598 | 0.8455 | 0.6465 |
| rs2389016 | 1.00E-08 | rs12065553 | 1 | 0.3443 | 0.3818 | 0.8636 | 0.3692 |
| rs2389016 | 1.00E-08 | rs2389024 | 1 | 0.3371 | 0.3993 | 0.9061 | 0.3692 |
| rs2389016 | 1.00E-08 | rs2154298 | 1 | 0.35 | 0.4313 | 0.9571 | 0.3262 |
| rs2389016 | 1.00E-08 | rs12118987 | 1 | 0.4025 | 0.5497 | 0.9244 | 0.3312 |
| rs2389016 | 1.00E-08 | rs10158964 | 1 | 0.3493 | 0.4594 | 0.839 | 0.2491 |
| rs2389016 | 1.00E-08 | rs1937787 | 1 | 0.434 | 0.5931 | 0.8752 | 0.2543 |
| rs2389016 | 1.00E-08 | rs3856038 | 0.965 | 0.4188 | 0.6911 | 0.9654 | 0.2772 |
| rs2389016 | 1.00E-08 | rs10493662 | 0.964 | 0.3594 | 0.3923 | 0.8493 | 0.3774 |
| rs4261101 | 1.00E-08 | rs4453022 | 0.963 | 0.2519 | 0.5699 | 0.3363 | 0.215 |
| rs12958048 | 3.60E-11 | rs4468713 | 0.858 | 0.569 | 0.7404 | 0.05335 | 0.2768 |
| rs4074723 | 3.10E-08 | rs4511370 | 0.869 | 0.6151 | 0.7961 | 0.84 | 0.9681 |
| rs7430565 | 2.90E-09 | rs6774461 | 1 | 0.1329 | 0.7494 | 0.1807 | 0.105 |
| rs7430565 | 2.90E-09 | rs2682405 | 0.967 | 0.1807 | 0.812 | 0.2691 | 0.1636 |
| rs7430565 | 2.90E-09 | rs7643792 | 0.967 | 0.1663 | 0.8058 | 0.2697 | 0.184 |
| rs7430565 | 2.90E-09 | rs1213048 | 0.967 | 0.1704 | 0.8108 | 0.2706 | 0.1827 |
| rs7430565 | 2.90E-09 | rs9857883 | 0.967 | 0.2292 | 0.8517 | 0.2798 | 0.2048 |
| rs11135349 | 1.10E-09 | rs10866752 | 0.81 | 0.3304 | 0.3124 | 0.3151 | 0.6401 |
| rs4869056 | 6.80E-09 | rs11747772 | 0.931 | 0.3579 | 0.3431 | 0.5339 | 0.4227 |
| rs4869056 | 6.80E-09 | rs11738110 | 0.899 | 0.3959 | 0.3636 | 0.6622 | 0.5927 |
| rs4869056 | 6.80E-09 | rs883322 | 0.834 | 0.4268 | 0.2816 | 0.758 | 0.5952 |
| rs12666117 | 1.40E-08 | rs11561993 | 0.967 | 0.9872 | 0.2198 | 0.1912 | 0.06189 |
| rs12666117 | 1.40E-08 | rs12113865 | 0.837 | 0.7409 | 0.2728 | 0.4964 | 0.09299 |
| rs1354115 | 4.70E-09 | rs7044150 | 0.965 | 0.3715 | 0.4744 | 0.3247 | 0.1761 |
| rs1354115 | 4.70E-09 | rs4741790 | 0.965 | 0.2827 | 0.4369 | 0.4942 | 0.4155 |
| rs1354115 | 4.70E-09 | rs7033160 | 0.868 | 0.8874 | 0.606 | 0.5845 | 0.241 |
| rs1354115 | 4.70E-09 | rs4741798 | 0.801 | 0.8595 | 0.7233 | 0.4806 | 0.2342 |
| rs7856424 | 8.50E-09 | rs10759879 | 1 | 0.9186 | 0.3242 | 0.8277 | 0.6453 |
| rs61867293 | 7.00E-10 | rs11192270 | 0.865 | 0.7957 | 0.1699 | 0.303 | 0.7227 |
| rs61867293 | 7.00E-10 | rs10884071 | 0.826 | 0.8955 | 0.5312 | 0.5209 | 0.7894 |
| rs61867293 | 7.00E-10 | rs17766570 | 0.802 | 0.424 | 0.1119 | 0.4206 | 0.5602 |
| rs4904738 | 2.60E-09 | rs1983711 | 0.93 | 0.4879 | 0.1866 | 0.2595 | 0.9982 |
| rs915057 | 7.60E-10 | rs7229 | 0.966 | 0.7609 | 0.3643 | 0.7707 | 0.9363 |
| rs8025231 | 2.40E-12 | rs668644 | 0.846 | 0.6272 | 0.4287 | 0.3206 | 0.9017 |
| rs8025231 | 2.40E-12 | rs624991 | 0.818 | 0.6517 | 0.4061 | 0.306 | 0.8907 |
| rs8025231 | 2.40E-12 | rs8024814 | 0.815 | 0.6912 | 0.6527 | 0.3112 | 0.9395 |
| rs8025231 | 2.40E-12 | rs657586 | 0.815 | 0.7187 | 0.636 | 0.298 | 0.9228 |
| rs8025231 | 2.40E-12 | rs667471 | 0.815 | 0.7493 | 0.7686 | 0.29 | 0.9647 |
| rs7198928 | 1.00E-08 | rs11077203 | 1 | 0.2905 | 0.2424 | 0.1135 | 0.1331 |
| rs7198928 | 1.00E-08 | rs7192025 | 0.963 | 0.2471 | 0.2457 | 0.1627 | 0.09317 |

r= Correlation between SNP Wray et al, 2017 and Generation Scotland SNP, bolded SNPs overlap between Wray et al, 2017 and Generation Scotland

**For all subsequent figures: horizontal black line indicates Bonferroni threshold of *p* = 5×10^-08^**

**Figure S1a:** GWAS of MHVS in the control-MDD study design controlling for all covariates except medication usage

**
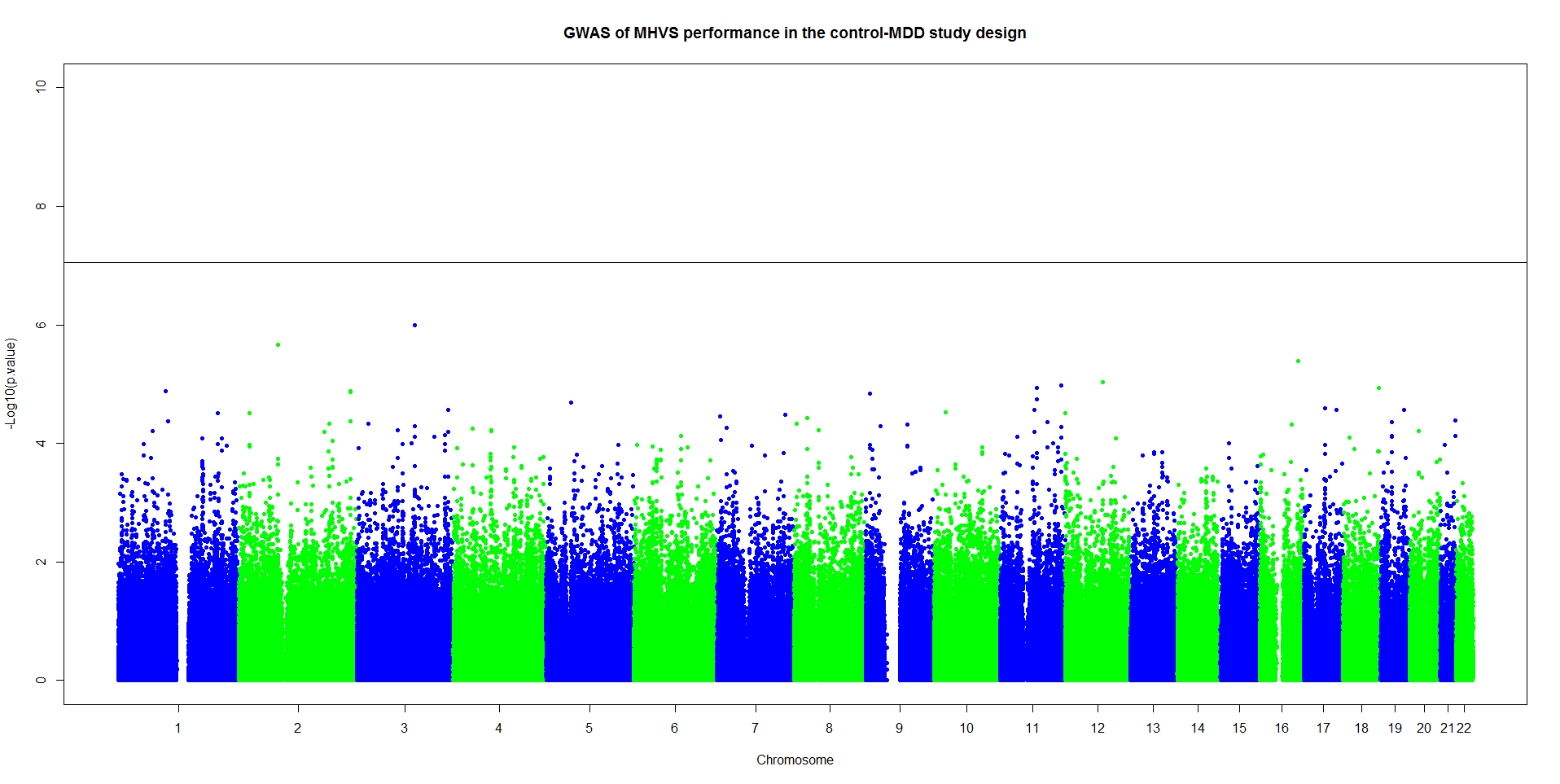
**

**Figure S1b:** GWAS of MHVS in the control-recurrent MDD study design controlling for all covariates except medication usage

**
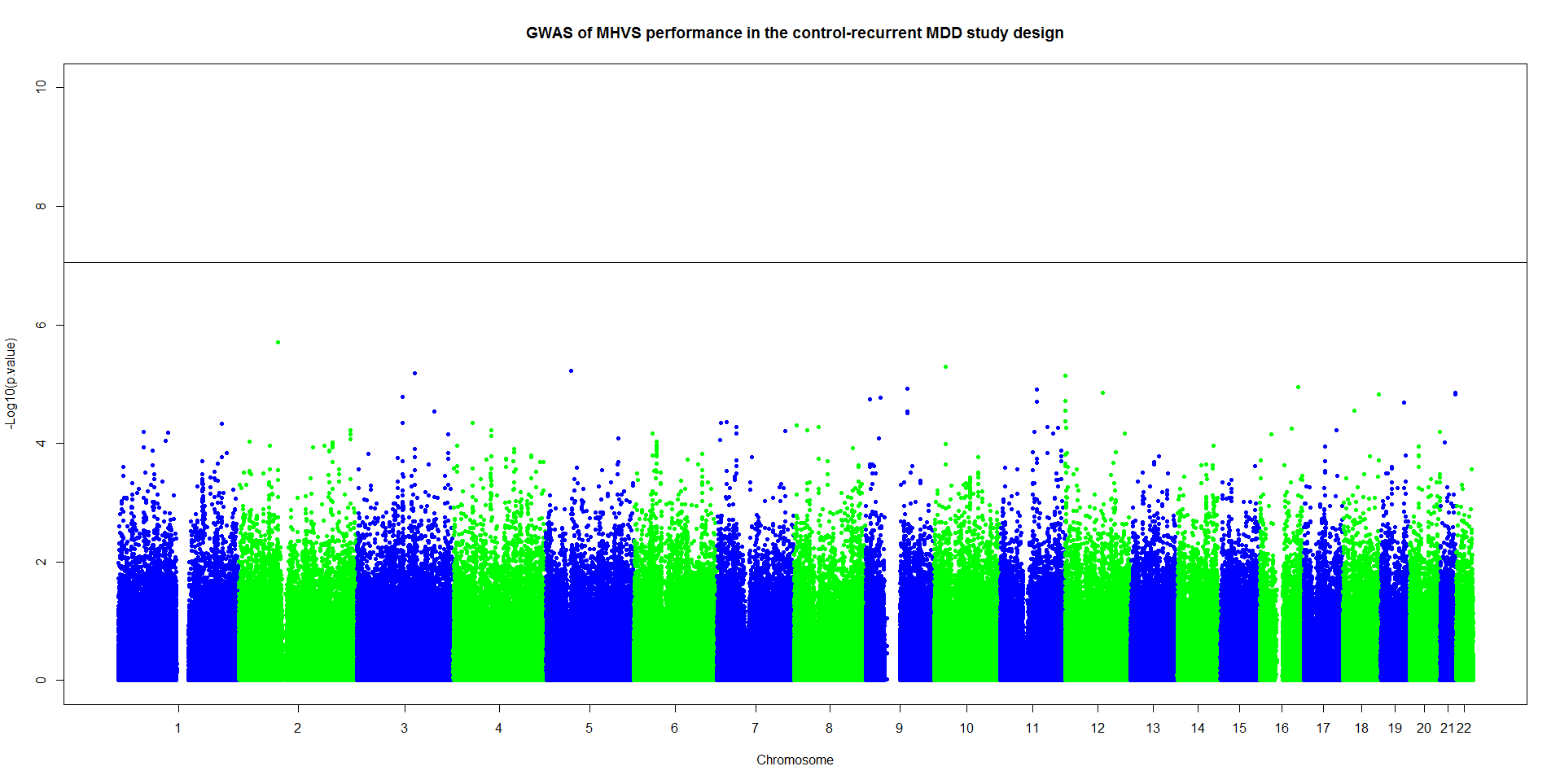
**

**Figure S2a:** GWEIS of MHVS in the control-MDD study design controlling for all covariates except medication usage

**
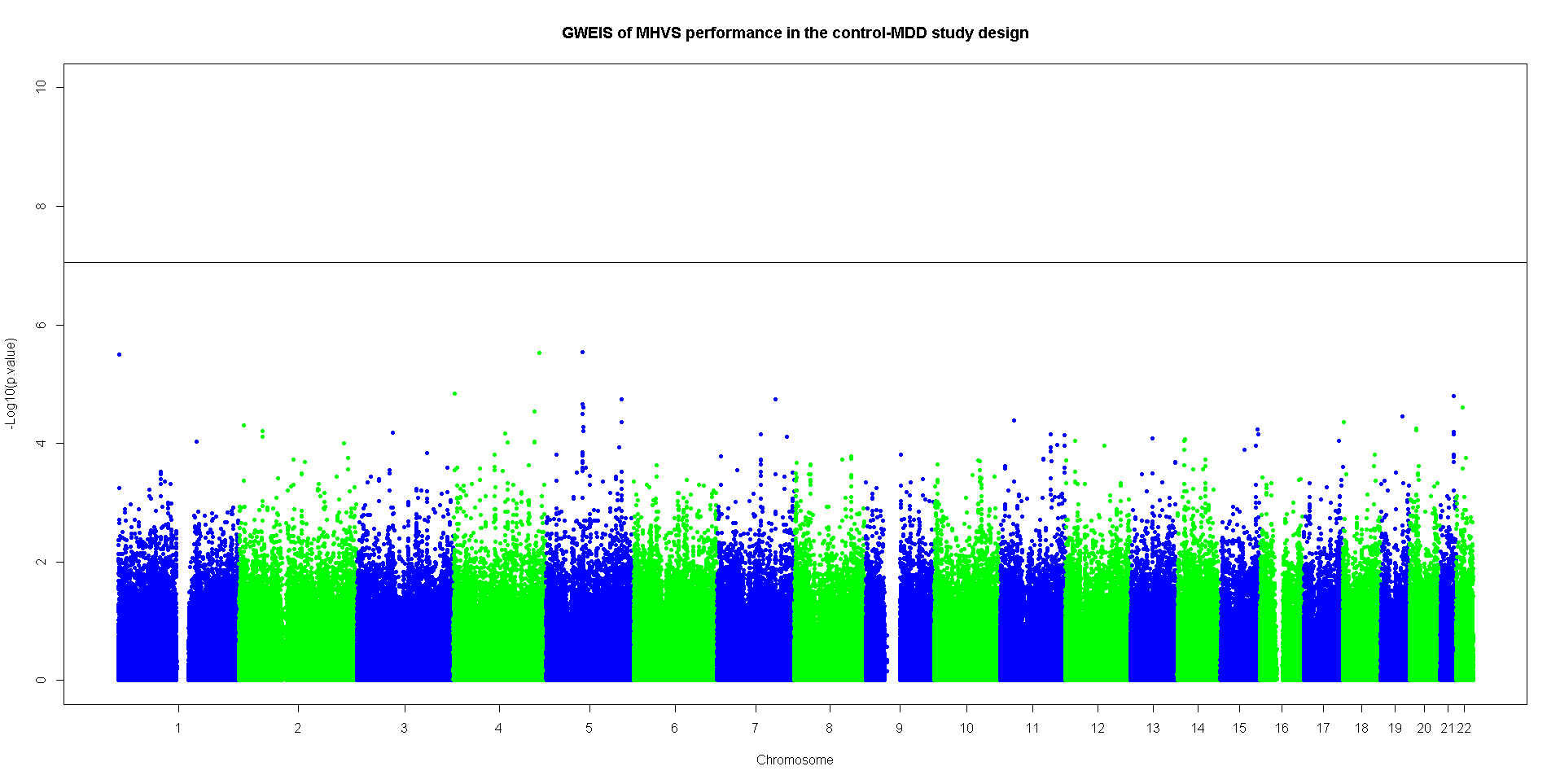
**

**Figure S2b:** GWEIS of MHVS in the control-recurrent MDD study design controlling for all covariates except medication usage

**
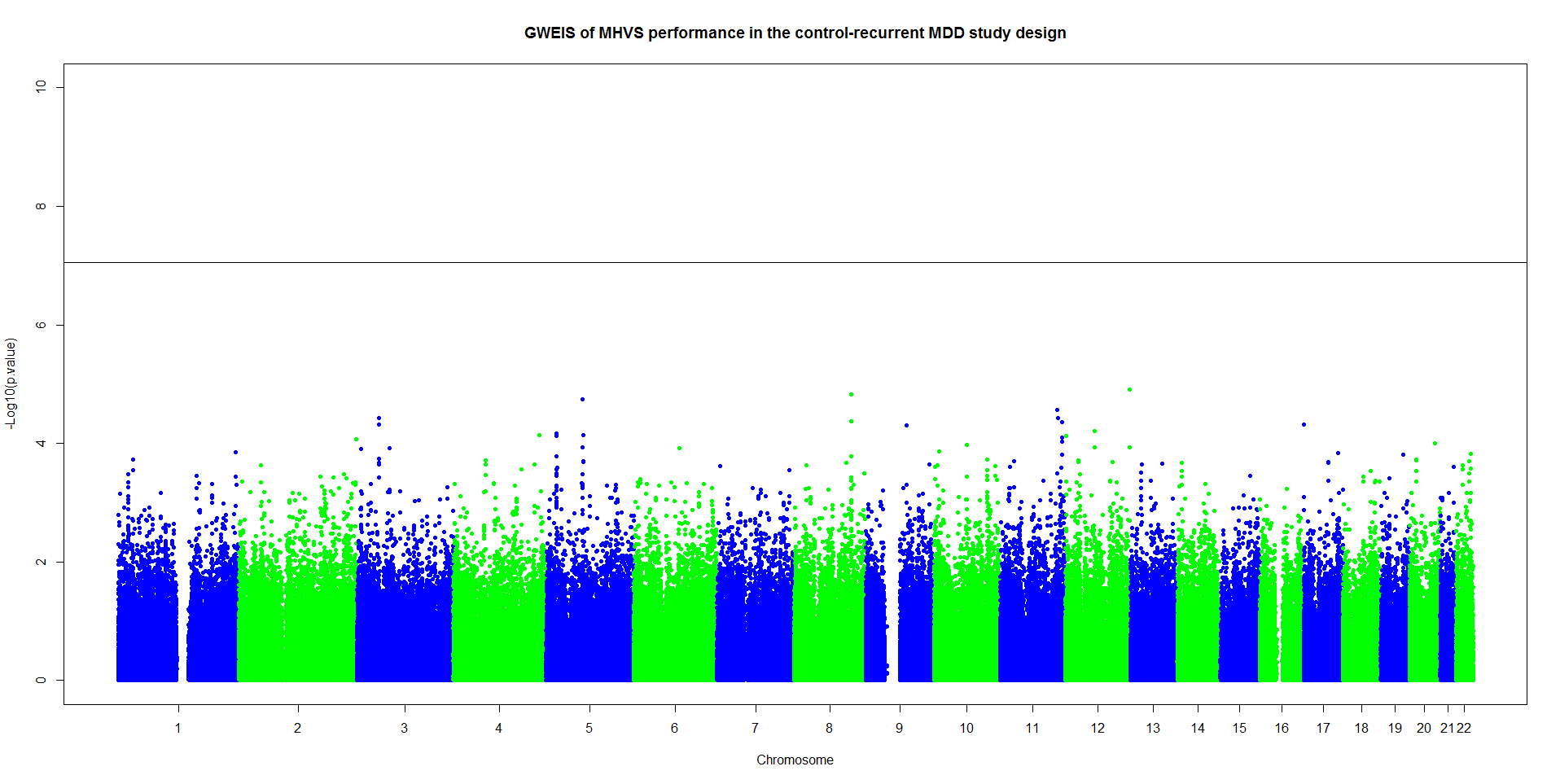
**

**Figure S3a:** GWAS of DST in the single-recurrent MDD study design controlling for all covariates except medication usage

**
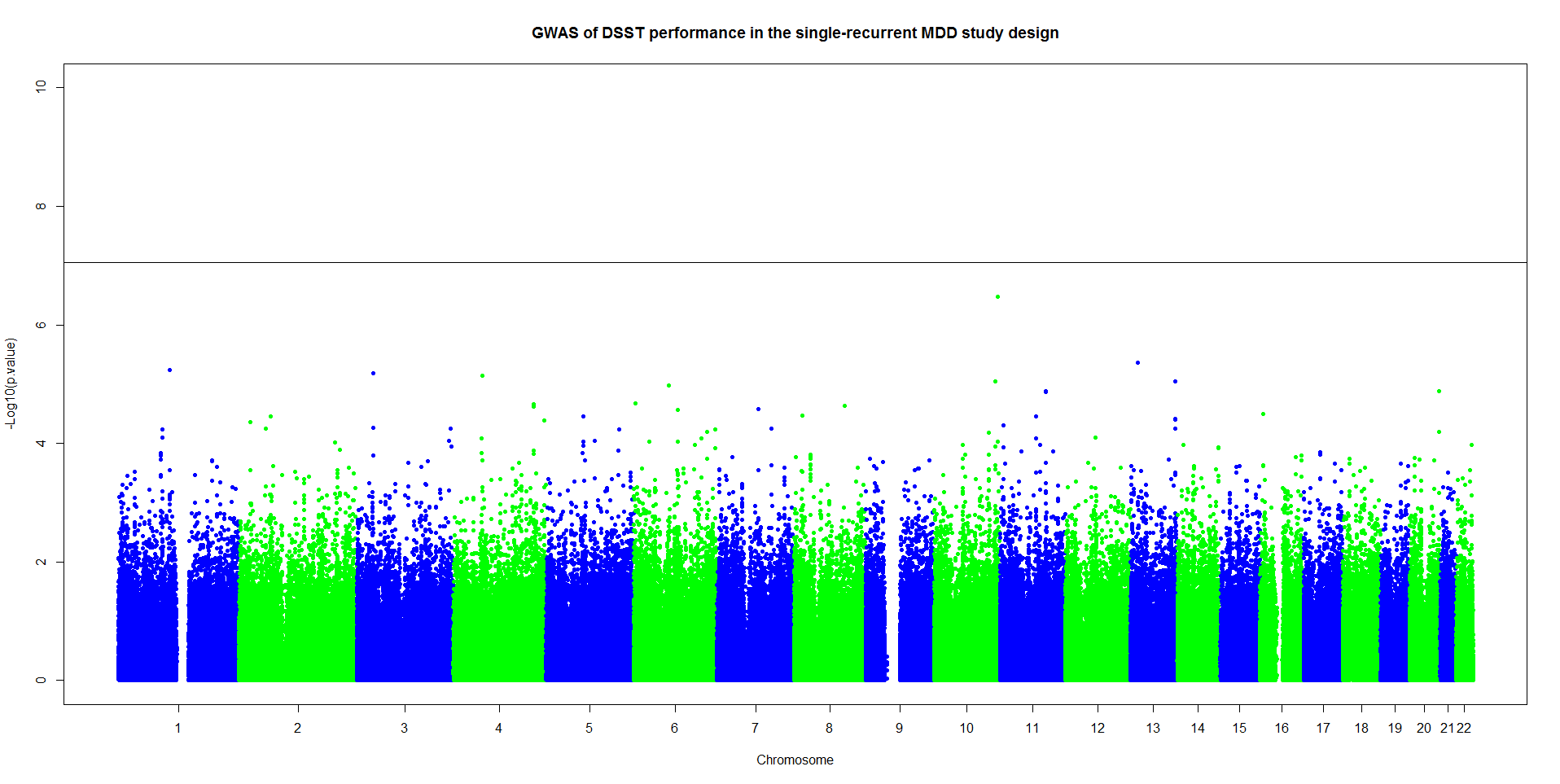
**

**Figure S3b:** GWAS of DST in the control-recurrent MDD study design controlling for all covariates except medication usage

**
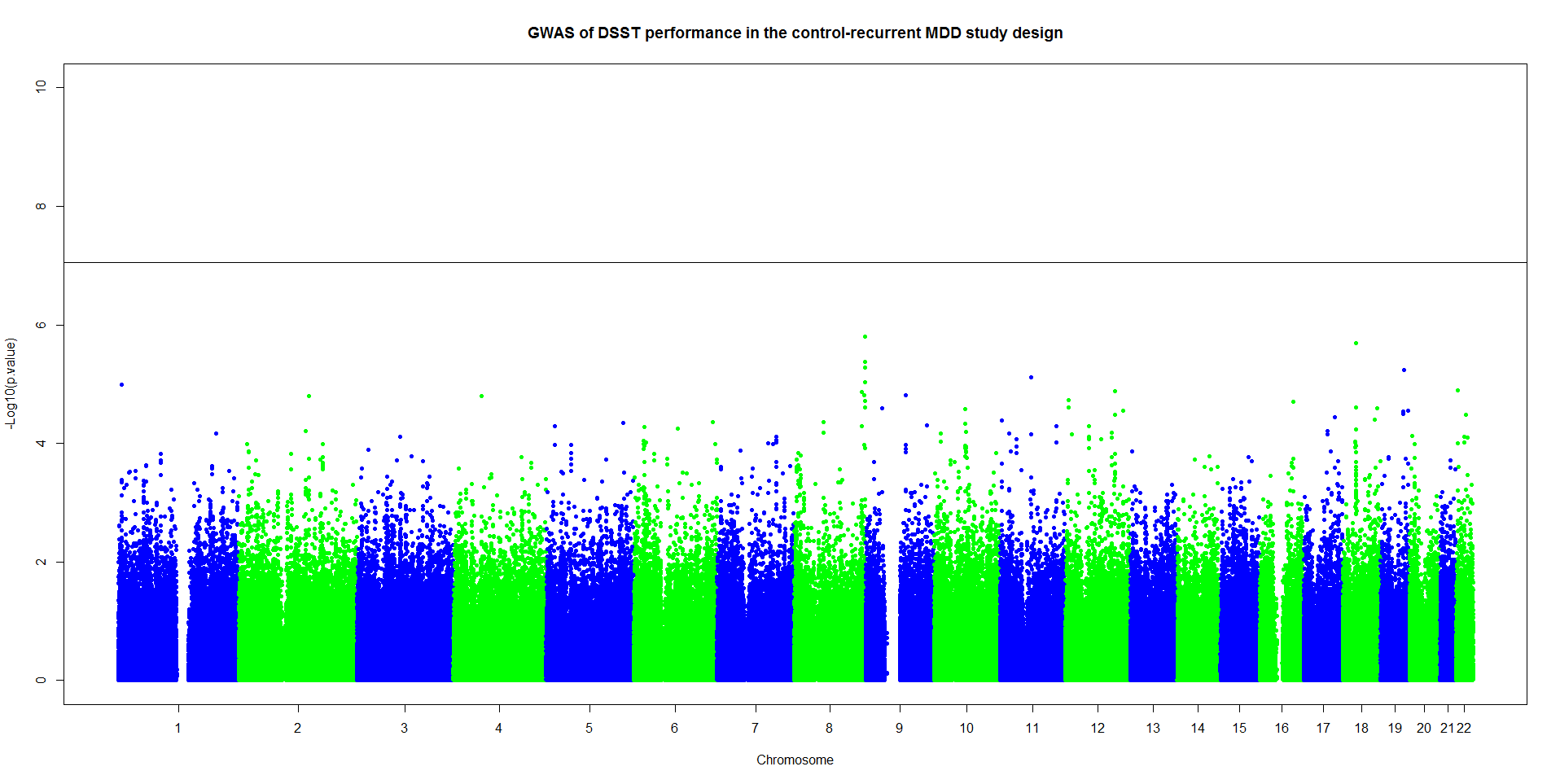
**

**Figure S4a:** GWEIS of DST in the single-recurrent MDD study design controlling for all covariates except medication usage

**
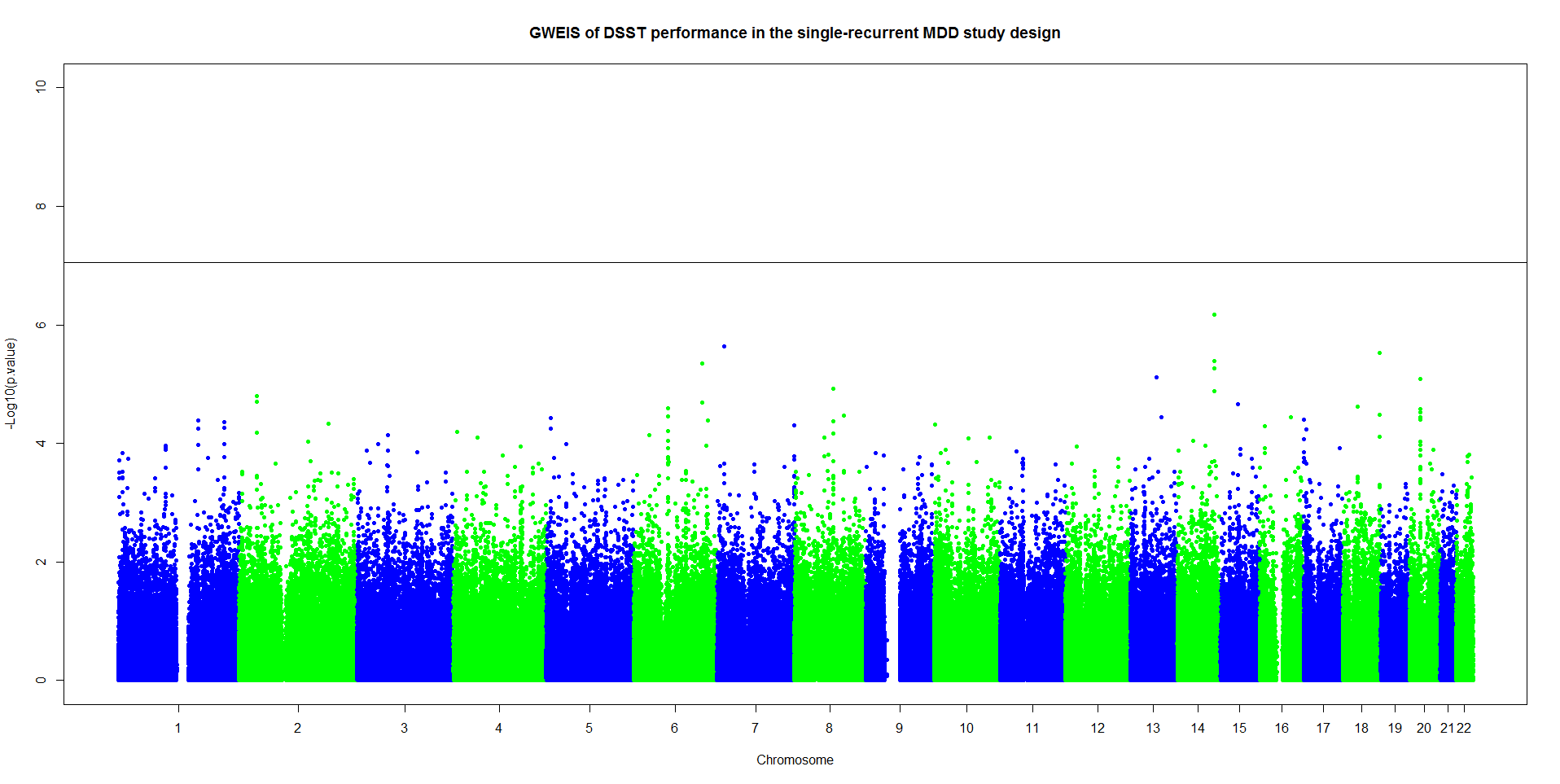
**

**Figure S4b:** GWEIS of DST in the control-recurrent MDD study design controlling for all covariates except medication usage

**
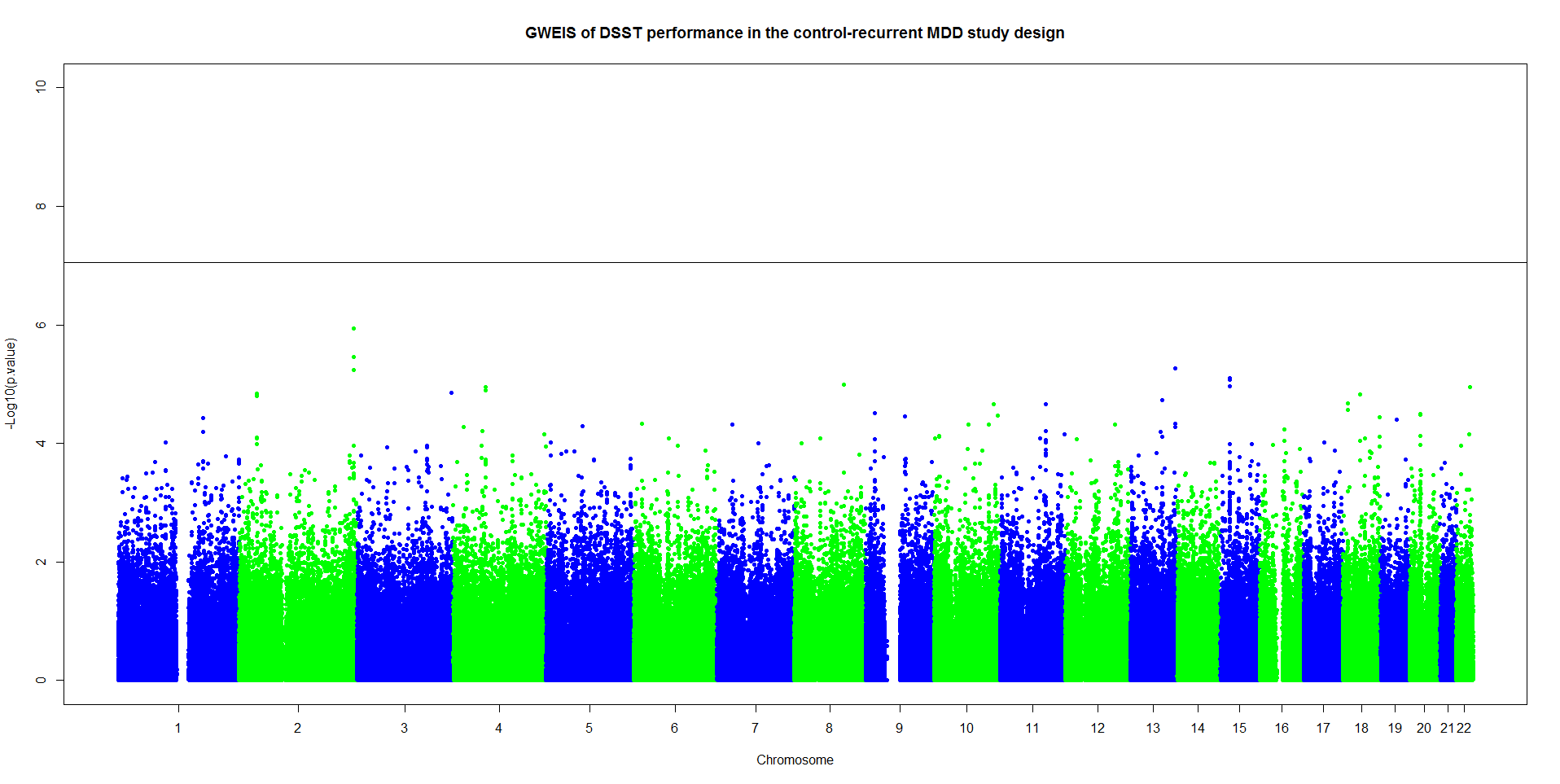
**

**Figure S5a:** GWAS of MHVS in the control-MDD study design controlling for all covariates

**
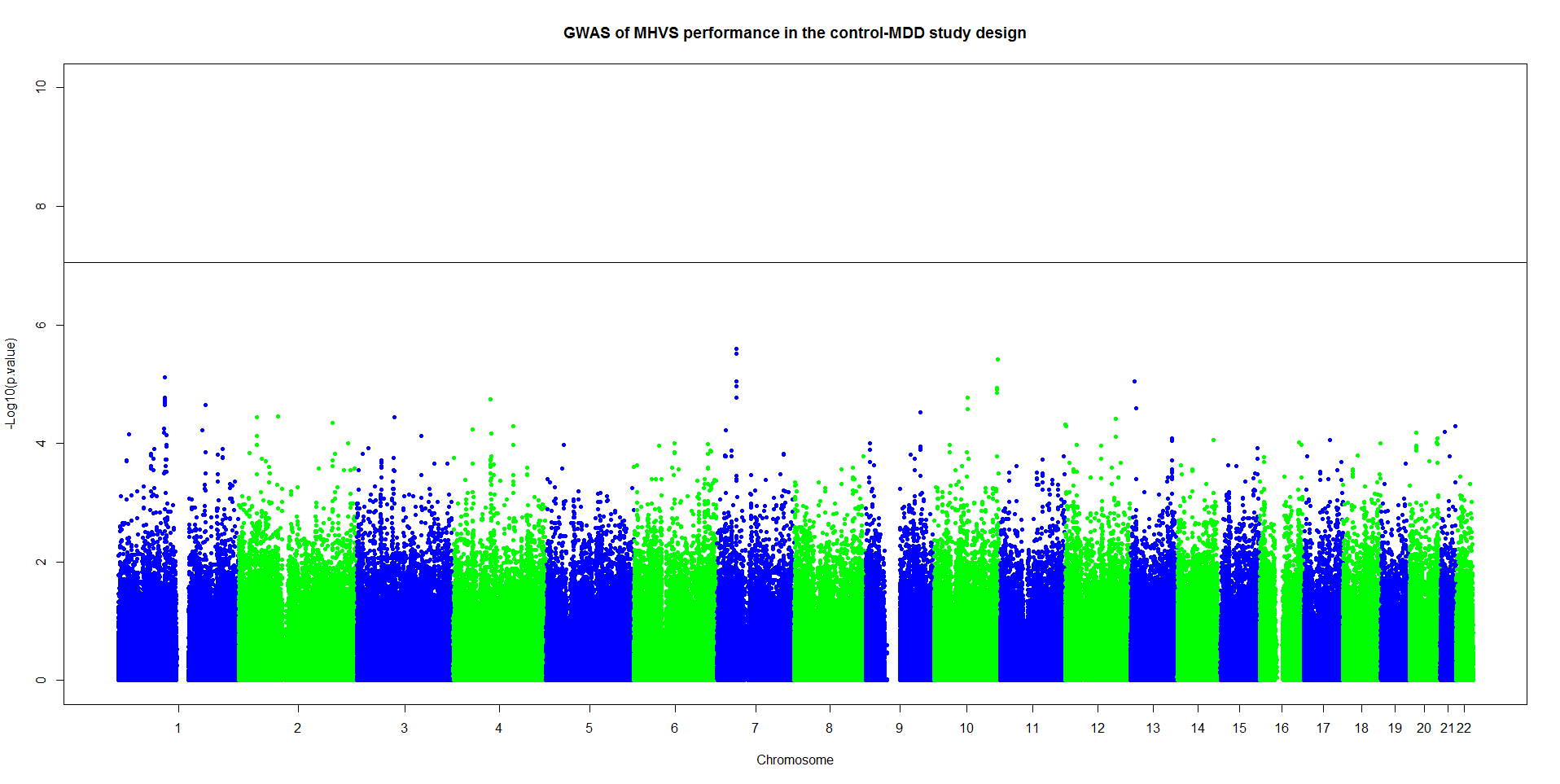
**

**Figure S5b:** GWAS of MHVS in the control-recurrent MDD study design controlling for all covariates

**
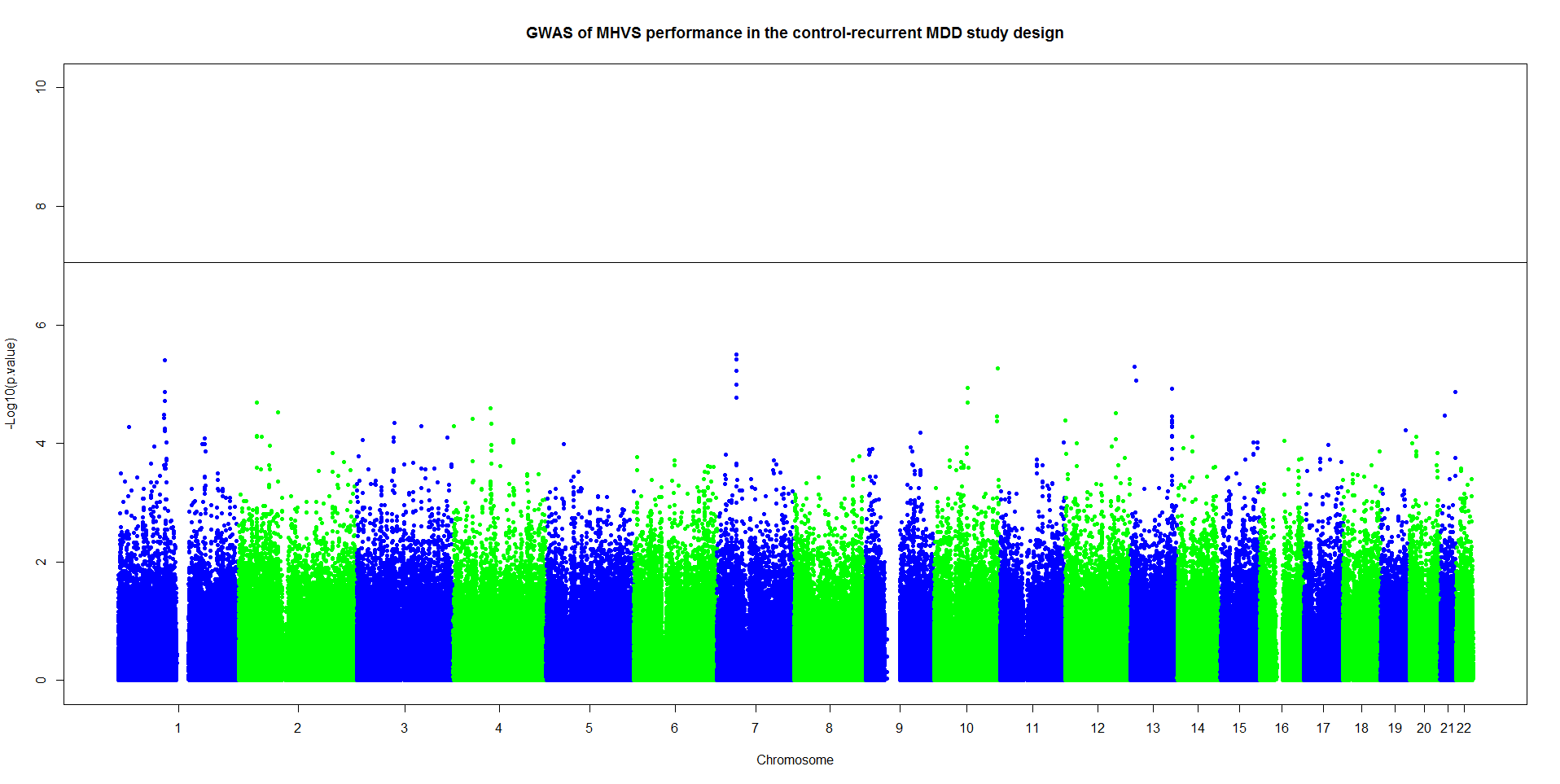
**

**Figure S6a:** GWEIS of MHVS in the control-MDD study design controlling for all covariates

**
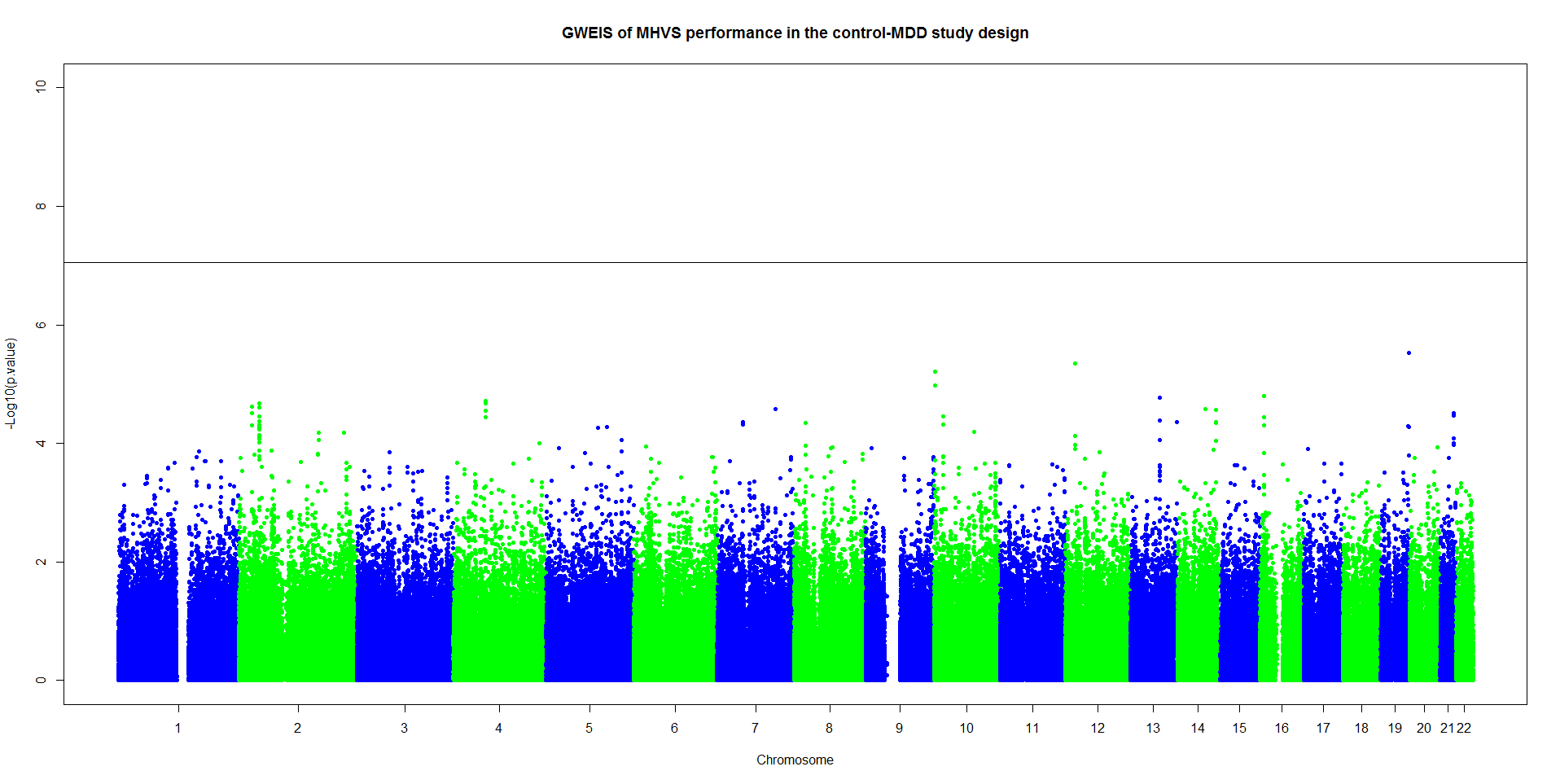
**

**Figure S6b:** GWEIS of MHVS in the control-recurrent MDD study design controlling for all covariates

**
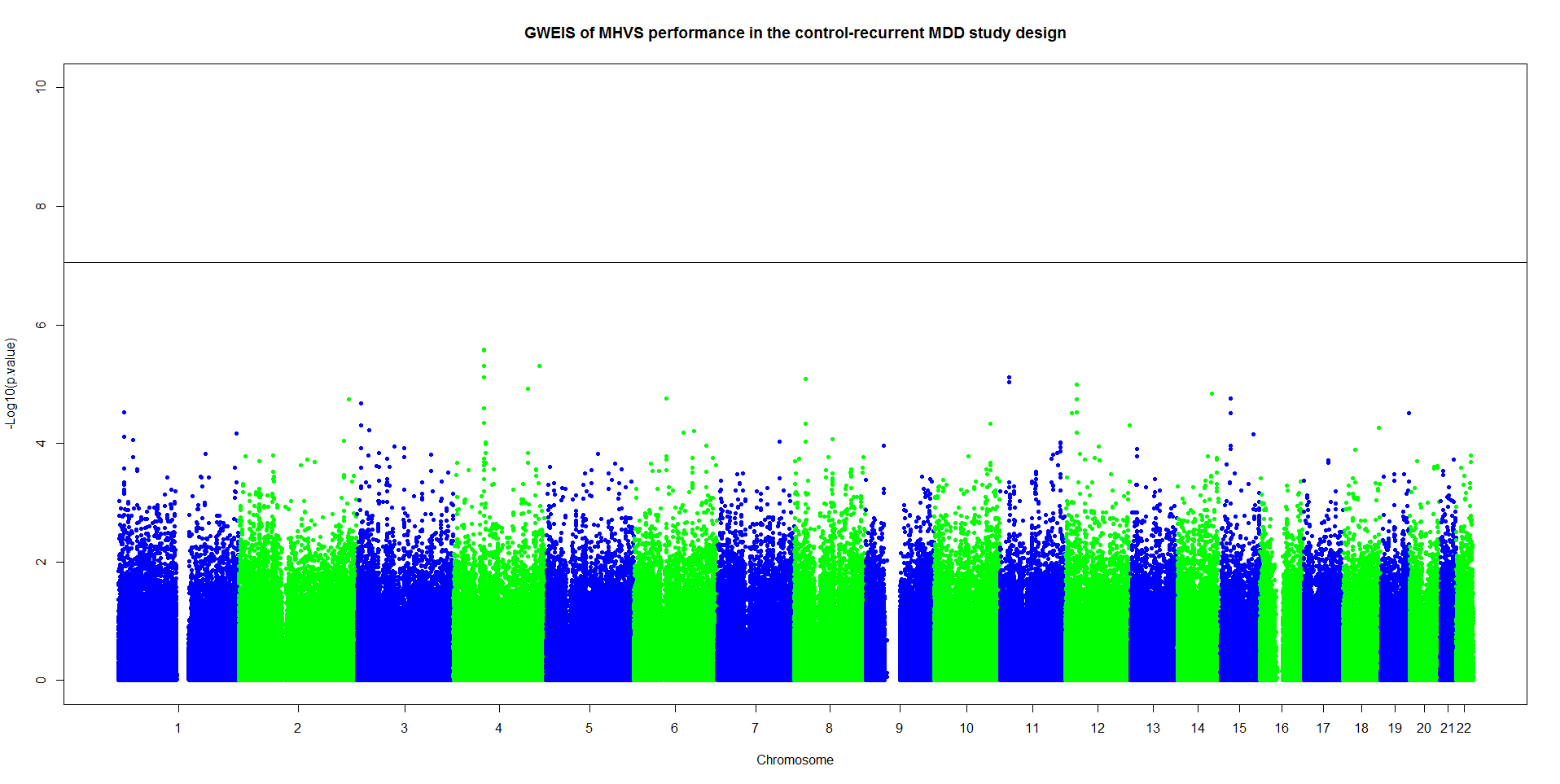
**
